# Unified Magnifying Endoscopic Classification (UMEC) of Gastrointestinal Lesions: A North American Validation Study

**DOI:** 10.1093/jcag/gwad055

**Published:** 2023-12-09

**Authors:** Mary Raina Angeli Fujiyoshi, Yusuke Fujiyoshi, Nikko Gimpaya, Robert Bechara, Thurarshen Jeyalingam, Natalia Causada Calo, Nauzer Forbes, Katarzyna Monika Pawlak, Kareem Khalaf, Rishad Khan, Michael Atalla, Akiko Toshimori, Yuto Shimamura, Mayo Tanabe, Christopher Teshima, Jeffrey D Mosko, Gary May, Haruhiro Inoue, Samir C Grover

**Affiliations:** Division of Gastroenterology, St. Michael’s Hospital, University of Toronto, 30 Bond Street, Toronto, Ontario, Canada, M5A 2H8; Digestive Diseases Center, Showa University Koto Toyosu Hospital, 5-1-38 Toyosu, Koto-ku, Tokyo, Japan 135-8577; Division of Gastroenterology, St. Michael’s Hospital, University of Toronto, 30 Bond Street, Toronto, Ontario, Canada, M5A 2H8; Digestive Diseases Center, Showa University Koto Toyosu Hospital, 5-1-38 Toyosu, Koto-ku, Tokyo, Japan 135-8577; Division of Gastroenterology, St. Michael’s Hospital, University of Toronto, 30 Bond Street, Toronto, Ontario, Canada, M5A 2H8; Division of Gastroenterology, Queen’s University, Sydenham 4, 166 Brock Street, Kingston, Ontario, Canada K7L 5G2; Division of Gastroenterology, University Health Network, University of Toronto, 200 Elizabeth Street, Toronto, Ontario, Canada M5G 2C4; Division of Gastroenterology, St. Michael’s Hospital, University of Toronto, 30 Bond Street, Toronto, Ontario, Canada, M5A 2H8; Division of Gastroenterology, University of Calgary, 3280 Hospital Drive NW, Calgary, Alberta, Canada T2N 4Z6; Division of Gastroenterology, St. Michael’s Hospital, University of Toronto, 30 Bond Street, Toronto, Ontario, Canada, M5A 2H8; Division of Gastroenterology, St. Michael’s Hospital, University of Toronto, 30 Bond Street, Toronto, Ontario, Canada, M5A 2H8; Division of Gastroenterology, St. Michael’s Hospital, University of Toronto, 30 Bond Street, Toronto, Ontario, Canada, M5A 2H8; Division of Gastroenterology, St. Michael’s Hospital, University of Toronto, 30 Bond Street, Toronto, Ontario, Canada, M5A 2H8; Digestive Diseases Center, Showa University Koto Toyosu Hospital, 5-1-38 Toyosu, Koto-ku, Tokyo, Japan 135-8577; Digestive Diseases Center, Showa University Koto Toyosu Hospital, 5-1-38 Toyosu, Koto-ku, Tokyo, Japan 135-8577; Digestive Diseases Center, Showa University Koto Toyosu Hospital, 5-1-38 Toyosu, Koto-ku, Tokyo, Japan 135-8577; Division of Gastroenterology, St. Michael’s Hospital, University of Toronto, 30 Bond Street, Toronto, Ontario, Canada, M5A 2H8; Division of Gastroenterology, St. Michael’s Hospital, University of Toronto, 30 Bond Street, Toronto, Ontario, Canada, M5A 2H8; Division of Gastroenterology, St. Michael’s Hospital, University of Toronto, 30 Bond Street, Toronto, Ontario, Canada, M5A 2H8; Digestive Diseases Center, Showa University Koto Toyosu Hospital, 5-1-38 Toyosu, Koto-ku, Tokyo, Japan 135-8577; Division of Gastroenterology, St. Michael’s Hospital, University of Toronto, 30 Bond Street, Toronto, Ontario, Canada, M5A 2H8

**Keywords:** diagnosis, endoscopy, magnifying NBI

## Abstract

**Background and study aim:**

Magnifying endoscopy enables the diagnosis of advanced neoplasia throughout the gastrointestinal tract. The unified magnifying endoscopic classification (UMEC) framework unifies optical diagnosis criteria in the esophagus, stomach, and colon, dividing lesions into three categories: non-neoplastic, intramucosal neoplasia, and deep submucosal invasive cancer. This study aims to ascertain the performance of North American endoscopists when using the UMEC.

**Methods:**

In this retrospective cohort study, five North American endoscopists without prior training in magnifying endoscopy independently diagnosed images of gastrointestinal tract lesions using UMEC. All endoscopists were blinded to endoscopic findings and histopathological diagnosis. Using histopathology as the gold standard, the endoscopists’ diagnostic performances using UMEC were evaluated.

**Results:**

A total of 299 lesions (77 esophagus, 92 stomach, and 130 colon) were assessed. For esophageal squamous cell carcinoma, the sensitivity, specificity, and accuracy ranged from 65.2% (95%CI: 50.9–77.9) to 87.0% (95%CI: 75.3–94.6), 77.4% (95%CI: 60.9–89.6) to 96.8% (95%CI: 86.8–99.8), and 75.3% to 87.0%, respectively. For gastric adenocarcinoma, the sensitivity, specificity, and accuracy ranged from 94.9% (95%CI: 85.0–99.1) to 100%, 52.9% (95%CI: 39.4–66.2) to 92.2% (95%CI: 82.7–97.5), and 73.3% to 93.3%. For colorectal adenocarcinoma, the sensitivity, specificity, and accuracy ranged from 76.2% (95%CI: 62.0–87.3) to 83.3% (95%CI: 70.3–92.5), 89.7% (95%CI: 82.1–94.9) to 97.7% (95%CI: 93.1–99.6), and 86.8% to 90.7%. Intraclass correlation coefficients indicated good to excellent reliability.

**Conclusion:**

UMEC is a simple classification that may be used to introduce endoscopists to magnifying narrow-band imaging and optical diagnosis, yielding satisfactory diagnostic accuracy.

## Introduction

Magnification endoscopy (ME) and magnifying narrow-band imaging (ME–NBI) are image-enhanced endoscopy technologies that may allow for the diagnosis of advanced neoplasia in the gastrointestinal (GI) tract on the basis of imaging characteristics.^1^ These technologies are commonly utilized in Japan and East Asia but are nascent in North America.^2^ Several diagnostic classification systems for image-enhanced endoscopy have been developed for different GI organs. These have been unified into three commonly used classifications in Japan: the Japan Endoscopy Society classification for the esophagus,^3^ the Magnifying Endoscopy Simple Diagnostic Algorithm for Early Gastric Cancer (MESDA-G),^4^ and the Japan NBI Expert Team (JNET) classification for colonic lesions.^5^

Recently, Inoue et al. developed the unified magnifying endoscopic classification (UMEC).^6^ This diagnostic classification system unified the criteria for the esophagus, stomach, and colon. UMEC divides optical diagnosis into one of the three categories: non-neoplastic, intramucosal neoplasia, and deep submucosal invasive cancer. A study assessing the performance of UMEC for optical diagnosis in the esophagus, stomach and colon was promising,^6^ additionally suggesting that UMEC is a simple optical diagnostic rubric. UMEC does not aim to replace existing organ-specific classifications utilized by expert endoscopists; however, a generalized use of an ME-NBI diagnostic criteria common to the GI tract may be more practical for non-expert endoscopists and general gastroenterologists.

There is limited literature on the performance of North American endoscopists on ME and ME-NBI optical diagnostic tools since the technology is emerging in this geographic market. The objective of this study is to ascertain the performance of North American endoscopists when using the UMEC.

## Methods

### Study design and population

This was a retrospective cohort study. A data set of ME-NBI images pre-collected from a single tertiary referral center, Showa University Koto Toyosu Hospital, was assessed. Endoscopic images from patients who underwent ME-NBI examination of the esophagus, stomach, and colon were reviewed by five North American endoscopists.

### Educational training

Five North American endoscopists (>1,000 procedures) without prior training in ME or image interpretation were recruited. The endoscopists were trained on the use of UMEC via a 10-min training video with examples of each element of UMEC from esophagus, stomach, and colon. Following completion of the training video, the endoscopists were given a post-test consisting of 10 total images from esophagus, stomach, and colon, and were required to correctly use UMEC prior to proceeding with the study.

### Image selection and data analysis

The data set included data on image quality as characterized by the initial endoscopist on a Likert scale for each image from 1 to 5, based on the clarity of the visual field and the microvascular structure (1 being the lowest quality: blurred/unclear visual field with obscured microvascularity, and 5 having the highest quality: clear visual field with no obstruction, clear microvascularity).

Two magnifying NBI images have been selected to represent pathology for each case and inserted into PowerPoint (Microsoft, Redmond, Washington, USA) presentation slides against a black background while preserving the file size, format, and image quality. Each case was assigned a number and subsequently arranged randomly according to random number tables created by Excel (Windows 2010; Microsoft).

Randomized ME-NBI images were then assessed separately by five North American endoscopists, trained as above. Each endoscopist scored each case on UMEC as 1, 2A, 2B, or 3 for esophagus and colon, or as 1A/2A vs. 2B/3 for stomach. The accuracy of UMEC-based diagnosis was assessed using the gold standard histopathology from the data set as a reference.

### Statistical analysis

Sensitivity, specificity, positive predictive value, negative predictive value, accuracy for diagnosis of three UMEC categories (non-neoplastic, intramucosal neoplasia, and deep submucosal invasive cancer) were calculated separately for each endoscopist. A two-way mixed effects intraclass correlation coefficient (ICC), with absolute agreement between the raters was used to estimate the interrater reliability. All analyses were performed using SPSS software version 29.0. (IBM Corp, Armonk, NY, USA).

### Ethical considerations

The study protocol adhered to the principles of the Declaration of Helsinki and was approved by the Ethics Committee of Showa University Koto Toyosu Hospital (IRB Registration No: 20T7016).

## Results

### Esophagus

A total of 77 cases were included in the study after excluding 40 which were designated as low-quality images. Population characteristics are shown in Table 1. Histopathological results showed the following: 9 normal, 12 inflammation, 20 intraepithelial neoplasia, and 36 SCC. The diagnostic performance was evaluated, and results are shown in Table 2. For esophageal squamous cell carcinoma, the sensitivity, specificity, and accuracy for all five endoscopists ranged from 65.2% (95% CI: 50.9–77.9) to 87.0% (95% CI: 75.3–94.6), 77.4% (95% CI: 60.9–89.6) to 96.8% (95% CI: 86.8–99.8), and 75.3% to 87.0%, respectively. The ICC was 0.875 (95% CI: 0.83-0.92) indicating good reliability.^7^ For the diagnosis of squamous neoplasms, the sensitivity, specificity, and accuracy for all five endoscopists ranged from 81.8 (95% CI: 71.4-89.8) to 97.0% (95% CI: 90.0–99.5), 54.5% (95% CI: 26.5–80.6) to 100%, and 79.2% to 93.5%, respectively. The ICC was 0.895 (95% CI: 0.85–0.93) indicating good reliability. For the diagnosis of deep submucosal cancer, the sensitivity, specificity, and accuracy for all five endoscopists were as follows: 85.7% (95% CI: 50.6–99.1), 77.1% (95% CI: 66.4–85.9) to 95.7% (95% CI: 89.3–98.9), and 77.9% to 94.8%, respectively. The ICC was 0.901 (95% CI: 0.86–0.93) indicating excellent reliability.

**Table 1.** Patients’ clinicopathological characteristics.

Variables	Esophagus (*n* = 77 )	Stomach (*n* = 92)	Colon (*n* = 130)
Age (year), mean (SD)	65 (13)	75 (12)	65 (13)
Sex			
Male (%)	57 (74%)	69 (75%)	75(58%)
Female (%)	20 (26%)	23 (25%)	55(42%)
Macroscopic type of cancer lesions	0-I = 4 0-IIa = 7 0-IIb = 16 0-IIc = 9	Ip + IIa = 1 IIa = 11 IIa + IIc = 3 IIc = 24	Is = 14 Isp = 15 Ip = 1 IIa = 15 IIc = 1
Treatment of cancer lesions			
Endoscopic resection	48	39	110
Surgery	5	0	20
Lesion diameter (mm) of cancer lesions, median (range)	10 (5–22)	18 (10–30)	8 (5–39)
Histopathology	Normal = 9 Inflammatory change = 12 Intraepithelial neoplasia = 20 Squamous cell carcinoma = 36	Gastritis = 28 Adenoma = 25 Adenocarcinoma = 39 –Well-differentiated tubular = 27 –Moderately differentiated tubular = 12	Hyperplastic polyp = 32 Low-grade dysplasia = 52 High-grade dysplasia =10 Adenocarcinoma = 36
Invasion depth			
Intramucosal	EP = 17/; LPM = 5/; MM = 4	31	5
Shallow submucosal	8	3	8
Deep submucosal	2	5	23

Abbreviations: EP, epithelium; LPM, lamina propria mucosa; MM, muscularis mucosa; SD, standard deviation.

**Table 2.** Diagnostic yield for esophageal lesions.

UMEC 1 vs. 2A + 2B + 3 (non-neoplastic vs. neoplastic)						
Endoscopist	Sensitivity % (95% CI)	Specificity % (95% CI)	PPV % (95% CI)	NPV % (95% CI)	Accuracy %	ICC
1	93.8 (86.3–98.0)	54.5 (26.5–80.6)	92.4 (84.4–97.2)	60.0 (30.0–85.4)	87.0	0.875 (0.83–0.92)
2	81.8 (71.4–89.8)	63.6 34.6–87.0)	93.1 (84.7–97.8)	36.8 (17.8–59.2)	79.2	
3	92.4 (84.4–97.2)	100	100	68.8 (44.5–87.5)	93.5	
4	97.0 (90.9–99.5)	72.7 (43.5–92.4)	95.5 (88.8–98.9)	80.0 (50.1–96.4)	93.5	
5	90.9 (82.4–96.3)	81.8 (53.7–96.7)	96.8 (90.0–99.5)	60.0 (35.1–81.7)	89.6	
UMEC 1 + 2A vs. 2B + 3 (non-cancer vs. cancer)						
Endoscopist	Sensitivity % (95% CI)	Specificity % (95% CI)	PPV % (95% CI)	NPV % (95% CI)	Accuracy %	ICC
1	82.2 (69.4 –91.5)	90.3 (76.8–97.5)	92.5 (81.7–98.1)	77.8 (62.6–89.2)	84.4	0.895 (0.85–0.93)
2	71.7 (57.8–83.3)	80.6 (64.6–91.8)	84.6 (71.3–93.6)	65.8 (50.0–79.5)	75.3	
3	65.2 (50.9–77.9)	96.8 (86.8–99.8)	96.8 (86.6–99.8)	65.2 (50.9–77.9)	77.9	
4	84.8 (72.6–93.2)	77.4 (60.9–89.6)	84.8 (72.6–93.2)	77.4 (60.9–89.6)	81.8	
5	87.0 (75.3–94.6)	87.1 (72.5–95.8)	90.9 (80.1–97.1)	81.8 (66.5–92.3)	87.0	
UMEC 1 + 2A + 2B vs. 3 (others vs. deep submucosal invasive cancer)						
Endoscopist	Sensitivity % (95% CI)	Specificity % (95% CI)	PPV % (95% CI)	NPV % (95% CI)	Accuracy %	ICC (95% CI)
1	85.7 (50.6–99.1)	91.3 (83.2–96.4)	50.0 (23.8–76.2)	98.4 (93.3–99.9)	90.9	0.901 (0.86–0.93)
2	85.7 (50.6–99.1)	82.9 (72.9–90.4)	33.3 (14.8–56.3)	98.3 (92.7–99.9)	83.1	
3	85.7 (50.6–99.1)	95.7 (89.3–98.9)	66.7 (34.5–90.5)	98.5 (93.7–99.9)	94.8	
4	85.7 (50.6–99.1)	77.1 (66.4–85.9)	27.3 (11.9–47.7)	98.2 (92.2–99.9)	77.9	
5	85.7 (50.6–99.1)	92.9 (85.3–97.4)	54.5 (26.5–80.6)	98.5 (93.5–99.9)	92.2	

Abbreviations: CI, confidence interval; ICC, intraclass correlation coefficient; NPV, negative predictive value; PPV, positive predictive value; UMEC, unified magnifying endoscopic classification.

### Stomach

A total of 92 gastric lesions were included in the study after excluding 58 low-quality images. Population characteristics are shown in Table 1. Histopathological results showed the following: 53 non-cancer and 39 cancer. For gastric adenocarcinoma, the sensitivity, specificity, and accuracy for all five endoscopists ranged from 94.9% (95% CI: 85.0–99.1) to 100%, 52.9% (95% CI: 39.4–66.2) to 92.2% (95% CI: 82.7–97.5), and 73.3% to 93.3%, respectively (Table 3). The ICC was 0.894 (95% CI: 0.85-0.93) indicating good reliability.

**Table 3.** Diagnostic yield for stomach lesions.

UMEC 1 + 2A vs. 2B + 3 (non-cancer vs. cancer)						
Endoscopist	Sensitivity % (95% CI)	Specificity % (95% CI)	PPV % (95% CI)	NPV % (95% CI)	Accuracy %	ICC
1	97.4 (89.2–99.9)	70.6 (57.3–81.9)	71.7 (58.7–82.6)	97.3 (88.6–99.8)	82.2	0.894 (0.85–0.93)
2	94.9 (85.0–99.1)	76.5 (63.7–86.6)	75.5 (62.4–86.1)	95.1 (85.7–99.2)	84.4	
3	94.9 (85.0– 99.1)	90.2 (80.1–96.4)	88.1 (76.1–95.6)	95.8 (87.7–99.3)	92.2	
4	100.0	52.9 (39.4–66.2)	61.9 (49.6–73.2)	100.0	73.3	
5	94.9 (85.0–99.1)	92.2 (82.7–97.5)	90.2 (78.8–96.9)	95.9 (87.9–99.3)	93.3	

Abbreviations: CI, confidence interval; ICC, intraclass correlation coefficient; NPV, negative predictive value; PPV, positive predictive value; UMEC, unified magnifying endoscopic classification.

### Colon

From 150 cases, a total of 130 cases were included in the study after excluding 20 low-quality images. Population characteristics are shown in Table 1. Histopathological results showed the following: 32 hyperplastic, 52 low-grade dysplasia, 10 high-grade dysplasia, and 36 adenocarcinomas. For colorectal adenocarcinoma, the sensitivity, specificity, and accuracy for all five endoscopists ranged from 76.2% (95% CI: 62.0–87.3) to 83.3% (95% CI: 70.3–92.5), 89.7% (95% CI: 82.1–94.9) to 97.7% (95% CI: 93.1–99.6), and 86.8% to 90.7%, respectively (Table 4). The ICC was 0.938 (95% CI: 0.92-0.95) indicating excellent reliability. For the diagnosis of colonic neoplasms, the sensitivity, specificity, and accuracy for all five endoscopists ranged from 90.7% (95% CI: 83.4–95.6) to 98.8% (95% CI: 95.0–99.), 83.7% (95% CI: 70.9–92.7) to 90.7% (95% CI: 79.7–97.0), and 88.4% to 93.0%, respectively. The ICC was 0.951 (95% CI: 0.94–0.96) indicating excellent reliability. For the diagnosis of deep submucosal invasive cancer, the sensitivity, specificity, and accuracy for all five endoscopists ranged from 45.8% (95% CI: 27.1–65.4) to 79.2% (95% CI: 60.4–92.0), 98.1% (95% CI: 94.2–99.7) to 100%, and 89.9% to 95.3%, respectively. The ICC was 0.877 (95% CI: 0.84-0.91) indicating good reliability.

**Table 4.** Diagnostic yield for colonic lesions.

UMEC 1 vs. 2A + 2B + 3 (non-neoplastic vs. neoplastic)						
Endoscopist	Sensitivity % (95% CI)	Specificity % (95% CI)	PPV % (95% CI)	NPV % (95% CI)	Accuracy %	ICC
1	96.5 (91.2–99.1)	86.0 (73.7–94.2)	93.3 (86.6–97.3)	92.5 (81.7–98.1)	93.0	0.938 (0.92–0.95)
2	90.7 (83.4–95.6)	83.7 (70.9–92.7)	91.8 (84.7–96.4)	81.8 (68.8–91.2)	88.4	
3	95.3 (89.5–98.5)	90.7 (79.7–97.0)	95.3 (89.5–98.5)	90.7 (79.7–97.0)	93.8	
4	98.8 (95.0–99.9)	72.1 (57.7–84.0)	87.6 (80.1–93.2)	96.9 (87.0–99.8)	89.9	
5	98.8 (95.0–99.9)	65.1 (50.3–78.2)	85.0 (77.2–91.1)	96.6 (85.7–99.8)	87.6	
UMEC 1 + 2A vs. 2B + 3 (non-cancer vs. cancer)						
Endoscopist	Sensitivity % (95% CI)	Specificity % (95% CI)	PPV % (95% CI)	NPV % (95% CI)	Accuracy %	ICC
1	83.3 (70.3–92.5)	94.3 (88.1–97.9)	87.5 (75.0–95.3)	92.1 (85.3–96.5)	90.7	0.951 (0.94–0.96)
2	76.2 (62.0–87.3)	97.7 (93.1–99.6)	94.1 (82.9–99.0)	89.5 (82.3–94.6)	90.7	
3	81.0 (67.4–90.8)	93.1 (86.5–97.2)	85.0 (71.9–93.7)	91.0 (83.9–95.8)	89.1	
4	81.0 (67.4–90.8)	89.7 (82.1–94.9)	79.1 (65.4–89.3)	90.7 (83.4–95.6)	86.8	
5	78.6 (64.7–89.1)	92.0 (85.0–96.5)	82.5 (68.9–92.1)	89.9 (82.5–95.0)	87.6	
UMEC 1 + 2A + 2B vs. 3 (others vs. deep submucosal invasive cancer)						
Endoscopist	Sensitivity % (95% CI)	Specificity % (95% CI)	PPV % (95% CI)	NPV % (95% CI)	Accuracy %	ICC (95% CI)
1	79.2 (60.4–92.0)	99.0 (95.9–99.9)	95.0 (79.8–99.7)	95.4 (90.4–98.3)	95.3	0.877 (0.84–0.91)
2	45.8 (27.1–65.4)	100.0	100.0	89.0 (82.5–93.8)	89.9	
3	58.3 (38.5–76.5)	99.0 (95.9–99.9)	93.3 (73.8–99.6)	91.2 (85.1–95.5)	91.5	
4	54.2 (34.6–72.9)	98.1 (94.2–99.7)	86.7 (64.2–97.7)	90.4 (84.0–94.9)	89.9	
5	54.2 (34.6–72.9)	99.0 (95.9–99.9)	92.9 (72.1–99.6)	90.4 (84.2–94.9)	90.7	

Abbreviations: CI, confidence interval; ICC, intraclass correlation coefficient; NPV, negative predictive value; PPV, positive predictive value; UMEC, unified magnifying endoscopic classification.

## Discussion

This study aimed to evaluate the performance of North American endoscopists when using the UMEC. To date, only one prior study has investigated the use of a Japan-based optical diagnostic tool among North American endoscopists as a single-center, single endoscopist study.^8^ The results of the present study demonstrated that UMEC is a simple classification to introduce North American endoscopists in ME-NBI and optical diagnosis, yielding satisfactory diagnostic accuracy for non-neoplastic, intramucosal neoplasia, and deep submucosal invasive cancer.

In applying ME-NBI, it is important to understand the differences in its role based on the organ. When applied to esophagus and colon, ME–NBI plays a role in analyzing the histopathology of squamous epithelium (esophagus) or columnar epithelium (colon) as well as cancer invasion depth. On the other hand, in the stomach, ME–NBI is used to analyze the histopathology of columnar epithelium to differentiate between non-cancer and cancer.

### Esophagus

**Figure 1.**
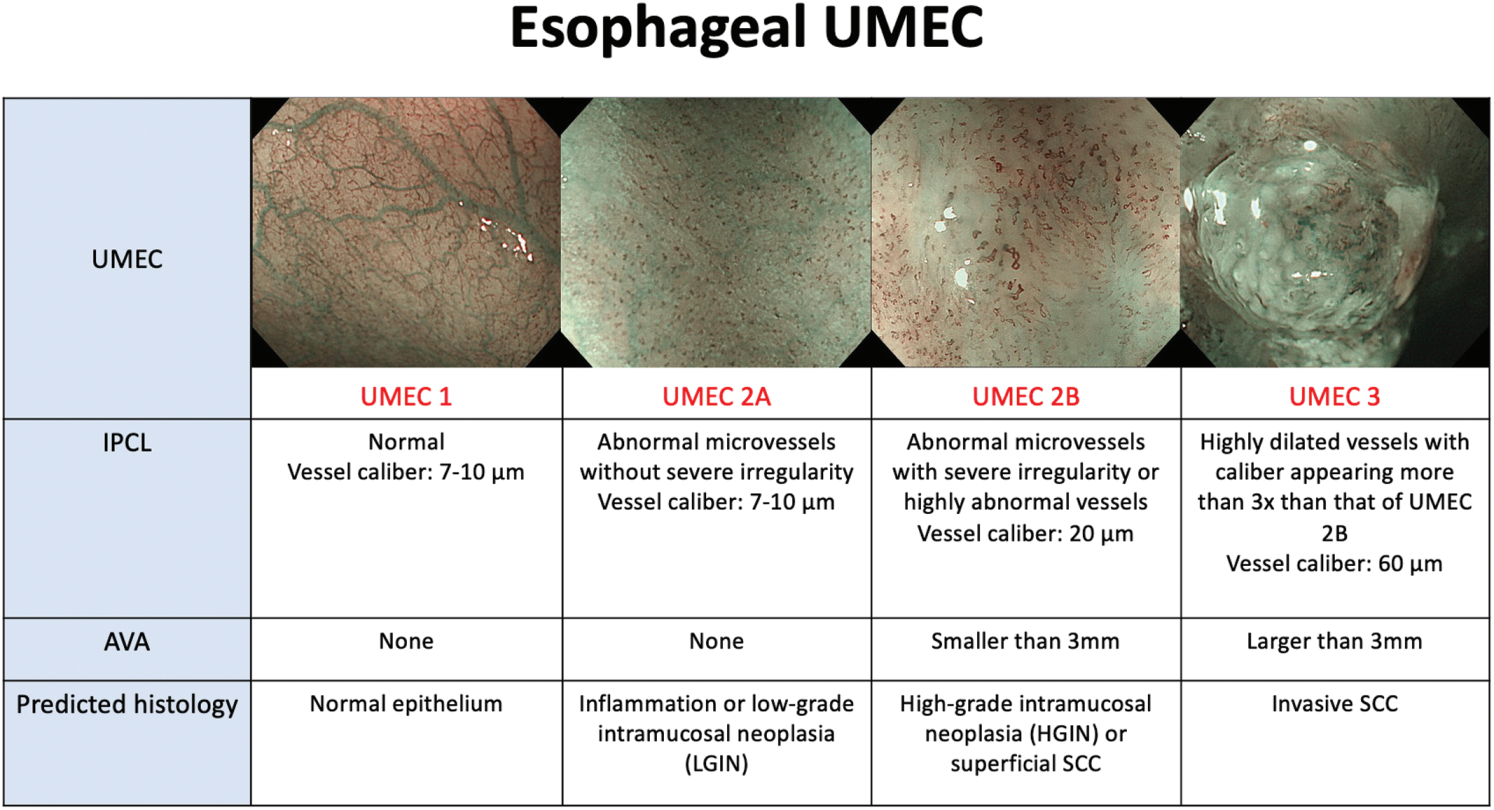
UMEC in the esophagus: Components included in the assessment are IPCL morphology and AVA.

In Japan, the use of the IPCL Pattern Classification to estimate histological atypia by magnifying NBI was introduced in 2007 by Inoue.^9^ Factors that were used to assess morphological changes of the IPCL pattern included dilation, tortuosity, and changes in caliber and shape. With this classification, accurate diagnosis of esophageal SCC has been demonstrated with rates showing more than 80%.^10–15^ Based on the feasibility pilot study of UMEC,^6^ we elected to focus on IPCL morphology and avascular area (AVA) (fig. 1), and the report showed more than 80% accurate diagnosis of esophageal SCC and more than 90% accurate diagnosis of deep submucosal invasion.

In the present study, the accuracy for the diagnosis of esophageal SCC ranged from 75.3% to 87.0% for all five North American endoscopists after being trained on the use of UMEC. The accuracy for the diagnosis of deep submucosal cancer ranged from 77.9% to 94.8% for all five endoscopists. These results appear to be satisfactory and comparable to previous studies in Japan despite that esophageal SCC is less encountered in North America. Although encountering a squamous lesion is not frequent in North America, using a simplified categorization similar to other organs such as UMEC may be more beneficial since there is no need to remember a different classification. In addition, considering Barrett’s esophagus and esophageal adenocarcinoma are more common than SCC in North America, expanding the UMEC to Barrett’s esophagus should be attempted in future studies.

### Stomach

**Figure 2.**
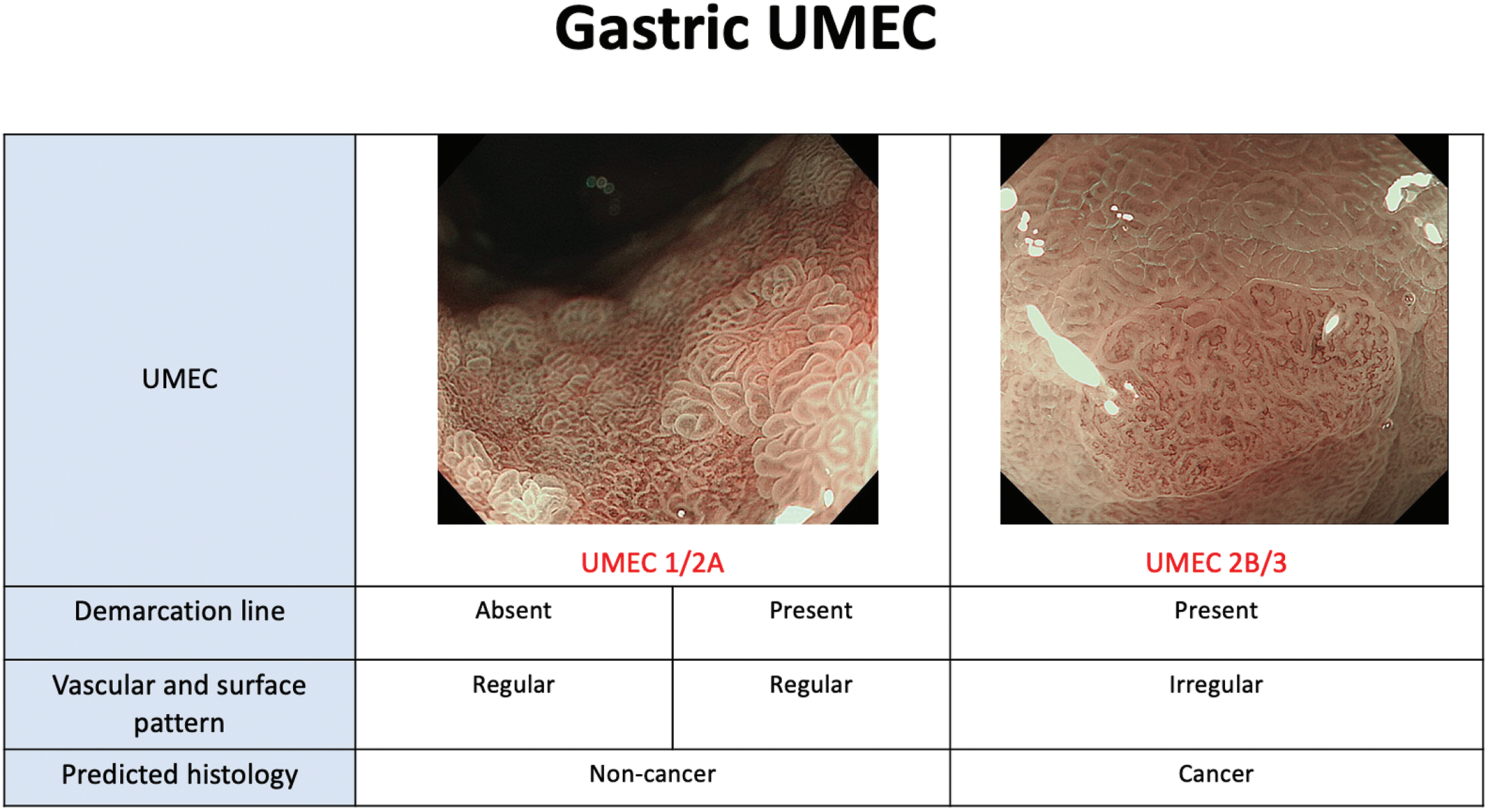
UMEC in the stomach: Components included in the assessment are demarcation line and vascular and surface pattern.

In Japan, the vascular surface (VS) classification was developed in 2009 by Yao as the standard method for the evaluation of gastric lesions.^16^ Factors assessed in this classification include the microvascular (MV) pattern and microsurface (MS) pattern which are assessed independently. According to this classification, typical findings of early gastric cancer include an identifiable demarcation line with irregular MV and MS patterns inside the demarcation line. By applying the same principles, the MESDA-G^4^ was developed as a diagnostic strategy for gastric mucosal cancer. These classifications suggest whether resection is required or not. With this classification and strategy, previous studies have shown satisfactory to excellent diagnostic accuracy rates (79% to >95%).^17–19^ UMEC (fig. 2) was based on the MESDA-G, therefore, the goal of UMEC in the stomach is not to guide therapeutic options but to indicate whether the lesion should be resected or not. The pilot study of UMEC showed an overall diagnostic accuracy of 89.5%.^6^ The pilot study also reported that due to lack of studies and sufficient evidence that non-neoplastic lesions can be distinguished from adenoma by image-enhanced magnifying endoscopy, UMEC 1 and 2A were not divided. Similarly, there was no sufficient evidence to conclude that image-enhanced magnifying endoscopy is clinically useful in diagnosing invasion depth, therefore, UMEC 2B and 3 were not divided.^20–24^

In the present study, the accuracy for the diagnosis of gastric adenocarcinoma ranged from 73.3% to 93.3% for all five endoscopists after being trained on the use of UMEC. These results appear to be satisfactory and comparable to previous studies, even though gastric adenocarcinoma is less encountered in North America compared to Asia.

### Colon

**Figure 3.**
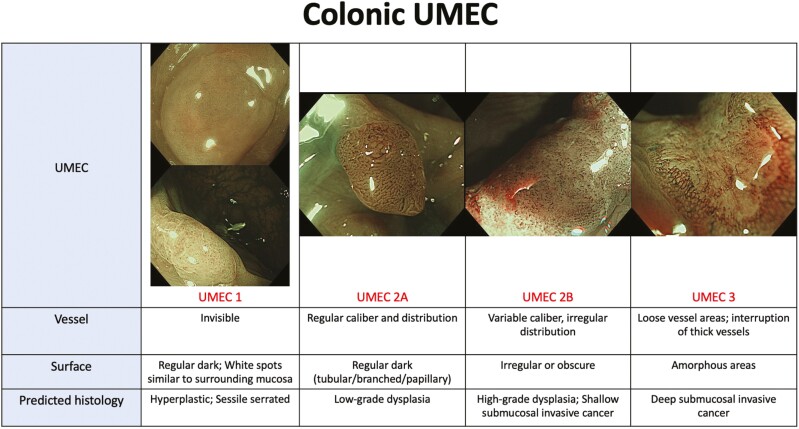
UMEC in the colon: Components included in the assessment are vessel pattern and surface pattern.

Globally, the classification being used most commonly for colorectal polyps is the NBI International Colorectal Endoscopic (NICE) Classification which was developed in 2012.^25,26^ Components used in assessment include color, vessels, and surface pattern. This classification has been widely accepted as a simple classification in applying NBI with or without optical magnification. In 2016, the JNET classification was developed to unify previous classifications and provide appropriate treatment strategies for each specific category. This classification is widespread and has been shown to have satisfactory diagnostic performance.^27,28^ UMEC (fig. 3) encompasses the JNET classification. The UMEC pilot study reported an overall diagnostic accuracy of 93.3% for colorectal adenocarcinomas.^6^

In the present study, the accuracy for the diagnosis of colorectal adenocarcinoma ranged from 86.8% to 90.7% for all five endoscopists after being trained on the use of UMEC. NICE classification is a non-magnifying NBI classification. The image set that was used in this study included only ME-NBI images, which may be unfamiliar to North American endoscopists as this technology is not prevalent.

### Limitations

This validation of UMEC among North American endoscopists must be interpreted within the context of the following limitations. First, this was a study to assess only two ME-NBI images per case retrospectively. In clinical settings, information about the lesion with white light images, non-magnifying NBI images as well as information on background mucosa are also obtained at the same time. The diagnostic ability of ME-NBI using UMEC would be improved with additional information in real clinical settings. Additionally, the sample size was small, and a larger number of lesions and endoscopists are needed in future studies. While the endoscopists recruited for this study lacked prior training in ME-NBI, they were familiar with previous classifications such as IPCL classification, VS classification, and NICE or JNET classification. Their interpretation of the images may have been influenced by their prior experience with these classifications. Furthermore, it is important to note that there can be variations in endoscopic examination and photo documentation practices even among different Western endoscopists. Specifically, differences may exist in the level of detail and thoroughness in examinations and the quality of photo documentation. As highlighted in the ESGE position statement on optical diagnosis training,^29^ there is a recognized need for more optical diagnosis training across various settings. In this study, we utilized a curated set of images taken by expert Japanese endoscopists, which exhibited the area of interest with close-up, high-resolution images of the microsurface, and microvascular patterns. However, it is essential to acknowledge that this representation may not accurately reflect the practices across all Western endoscopists. In real-life Western clinical settings, the accuracy of optical diagnosis may differ from our study results,^30^ suggesting that the practical accuracy of optical diagnosis using UMEC could vary across different Western clinical contexts. Thus, it is imperative to evaluate the utility of UMEC in clinical practice within various Western settings in future studies. Finally, the training video was developed by a North American endoscopist with extensive expertise in optical diagnosis but was not validated beyond this.

### Conclusion

UMEC is a simple and practical classification that can be used to introduce endoscopists to ME-NBI and optical diagnosis, yielding satisfactory diagnostic accuracy.

## Supplementary data

Supplementary data are available at *Journal of the Canadian Association of Gastroenterology* online.

gwad055_suppl_Supplementary_Materials

## Data Availability

The data supporting the findings of this study are available upon reasonable request. To access the data, interested researchers may contact the corresponding author Dr. Samir C Grover and submit a request outlining the specific data needed for their research purposes. The authors will review the requests and provide the data, in compliance with applicable ethical and legal regulations. Due to privacy and confidentiality concerns, the raw data cannot be publicly shared or made openly accessible. However, efforts will be made to facilitate data sharing in a manner that respects participant privacy and meets data protection requirements. Supplementary materials and additional information may be provided to aid in the interpretation and replication of the study results.
